# Dental Inlaying with Porcelain

**Published:** 1889-04

**Authors:** W. Storer How


ARTICLE VIII.

### DENTAL INLAYING WITH PORCELAIN.

In continuation of the subject of dental repair by the employment of porcelain inlays as described in the *Dental Cosmos* for July, 1881, it is to be said that there are further details of manipulation in the setting of the circular inlays, which will here be briefly presented.

For preparing the cavity in the tooth, the barrel burs were adopted as being the best instruments at that time obtainable by dentists for whom the operation was described and shown. Since then the writer has had made, by the manufacturing company with which he is connected, a set of nine inlay burs, which have straight, slightly tapering sides, and are fine cut on their ends and sides.

The primary preparation of the cavity may be done with any form of bur at hand for the purpose,—as, for example, a square end fissure bur, and the depth of the cavity be cautiously, yet judiciously determined to be as great as will be compatible with the continued life of the pulp.

The formation of such cavities in natural teeth which have been extracted, and the subsequent grinding of these into sections through the artificial cavity and the pulp-cavity, will afford an experience that will be useful in aiding one to judge how deep the cavities may safely be made in living teeth of like character.

When the primary cavity has been formed and is about to be enlarged by an inlay bur, great care must be taken to hold the engine hand-piece steady to enter the cavity at a proper angle, and to hold the bur so firmly that it cannot run away and mar the cavity margins. Then slowly yet constantly push the inlay bur down to the bottom of the cavity, being very careful not to chip off the edges of the cavity by either too great pushing force or by swerving to one side or the other. It is also necessary that the bur should run quite true in the hand-piece in order that a hole of true and equal taper and also a truly cylindrical hole may be formed.

Failure in any of these points will be made manifest in the finishing process, as this discloses the slightest superficial defects.

The inlay must likewise be ground to a true frustum, and this is a work of more difficulty than might at first be supposed, especially when it is considered that the taper of the inlay is to be made correspondent to the taper of the cavity. With this object in view I have made the grinder-rest here exhibited, consisting of a corundum slab-holder and hand-piece support, which, by set-screw adjustment, enables the operator to rest the nose of the hand-piece on the support at an inclination that will permit the rotating inlay to be held on the slab at such an angle as will impart the desired taper to the walls of the inlay. By this means an inlay can be made of a slightly increased taper in comparison with the inlay bur, and therefore the inlay will touch the cavity all around its superficial border, thus reducing the joining to its lowest degree. The tapers of the cavity and the inlay should, however, coincide as closely as possible, because it will often be necessary to cut down on one side or the other, and that will disclose any increasing separation of the inlay from the cavity.

In still further improvement of the accessories to this operation, brass mandrels have been made, and because they are impervious to the water used in grinding, the shellac will stick the inlays to the mandrels with greater firmness than to the wood points. Two of the mandrels are reversible, and a set of three mandrels serves for the set of four inlays. These have been given such sizes and shapes as were deemed suitable for the meeting of nearly all cases, but if there be an occasion for the conversion of a larger into a smaller size, this may best be done at the laboratory lathe by simultaneously running the engine and holding the revolving inlay again at the rotating lathe-wheel. As another means for rapid grinding, a corundum slab having a suitably tapered perforation may be employed; or the arbor-hole of a corundum wheel may be utilized; but neither of these modes seem on the whole preferable to that previously described. The two most difficult points in the manipulative process are the formation of a truly round and nearly cylindrical cavity, and the grinding of an exactly, corresponding inlay. Another difficulty is the preparation of a permanent cement of a color similar to that of the tooth and inlay. Concerning the permanency of zinc oxyphosphate cements, there is nothing new to be said, and with regard to the permanency of inlays set with this class of cements, there are no recorded data on which to base an estimate of the probabilities of inlay continuance in a given case. The most that can now be said is that some zinc oxyphosphates are enduring in the side cavities in some mouths, and that in nearly every instance its wastage, apart from abrasive or attritional wear, is in lines of concavity, leaving the marginal contact undisturbed. A reasonable inference, therefore, is that the thinner the margin the slower the wastage. Hence it is fair to expect that a very close approximation of the inlay margin to the cavity margin will insure a prolonged duration for the thin intervening septum of connective oxyphosphate. The recent publication of inlay articles has attracted the attention of practitioners who allege the setting of them in zinc plastic cements many years ago, and they can doubtless testify to the more or less staying qualities of the work. On the other hand, it may be confidently anticipated that a class of practitioners will declare that the zinc phosphates are ephemeral in all places in the teeth of all mouths, and that inlay work of that character will be disappointing. That, in fact, it should in no case be advocated, and needs no experimentation to verify its clearly inferential impermanence.

Nevertheless, the experienced dentist will recall cases which (as, for instance, the young lady who had an engagement with some other person than the dentist) he would have been glad to have repaired with an inconspicuous material even though it should certainly be renewed within the year.

It will, however, be conceded that, when the long-expected permanent plastic cement shall have arrived, this inlay process will be deemed an excellent mode of repair, as the samples of inlaid extracted teeth herewith submitted for inspection convincingly attest. No. 1 shows a left superior central having a large labial cavity, and No. 2 a like right central that had a similar cavity, which now has an inlay sat in zinc phosphate. This inlay was made from an old cavity-stopper, and is not so dense as the inlays now furnished, nor is its shade closely similar to that of the tooth; but the operation is obviously, in some respects, an advance upon anything hitherto brought before the profession, and so far as appearances go it would be difficult to excel the similarly inlaid lower right and left cuspids, Nos. 3 and 4, the latter being readily accepted, as it indeed has been, for a perfectly sound tooth.

The vertical section of a central shows the close fitting of the inlay walls with the cavity walls, even after the surface has been cut down considerably around the margins. It also shows the absence of any cement on the floor of the cavity, and that fact in part explains the close contact of the wall; a very essential element of success in the operation.

The color of the cement modifies the appearance of the inlay, which is more or less translucent, according as its shade is light or dark, and the colour of the cement should therefore harmonize with that of the inlay when that is like that of the tooth; but when, as must frequently occur, the tooth is not exactly matched by the inlay, this may be toned up or down by a suitably-colored cement, such as experience only will enable the dental artist to prepare.

A practically permanent inlay can be made with gutta-percha as a joining medium, and the sample No. 6 shows that a very close wall joint may be made, and the floor also covered, because the mode of manipulating the gutta-percha admits of the complete filling of the cavity.

In practice, the fitted inlay, some preparation of gutta-percha, as Hill's stopping, and the required instruments, are all made hot on a dry water heater, the cavity dried with warm air, the cavity walls thinly lined with the gutta-percha made soft enough to stick to the walls, the inlay put in with a pair of pliers, and quickly pressed home with the hot flat end of an instrument large enough to retain and impart to the warm inlay heat sufficient to completely soften the gutta-percha and cause all the surplus to exude around the inlay. A cold instrument is then immediately substituted for the hot one, and pressure continued until the normal temperature has resulted.

After a short interval to allow the thorough cooling of the gutta-percha, the surface of the inlay may be ground and polished, and the operation finished at that sitting. No. 7 is an example of a small inlay which has been darkened by a bluish shade of gutta-percha, but the joining is a mere line, and the operation is such as would prove permanent and not unsightly in the mouth.

Too great care cannot be taken not to overheat the gutta-percha and so darken it; nor to soil it or the inlay or cavity margins; nor to fail to completely surround the inlay with gutta-percha. Either of these errors will be discovered as a blemish when the polishing process shall be commenced.

No. 8, an upper left lateral in section, shows a close-fitting, yet imperfectly surrounded inlay set in gutta-percha, No. 9 likewise shows one imperfectly set in crude white gutta-percha. No. 10 is an upper right central, having a small inlay set in sandarac, but it, as well as copal and mastic, has a yellow tinge, which is deepened by the melting heat necessary to soften it sufficiently, and hence a darkening of the inlay results.

At the present writing, gutta-percha softened by heat appears to be the best obtainable joining material, all things being duly considered.

The uses of these inlays will not be confined to the circular form cavities previously described, but in many buccal and crown cavities which cannot be given a circular form, the inlay may be approximately conformed, and set in gutta-percha with very satisfactory results.

In large crown and compound cavities where the pulp is nearly exposed the inlays may be set in cement with their bases inward, and so serve as caps to the pulps. In these instances the cement should extend but half-way up the inlay sides, and the remainder of the cavity be filled with gold or amalgam.

Many cases will arise in which, by notching or otherwise grinding an inlay, it may occupy the bulk of a cavity and present a porcelain surface for mastication, thus in a great degree diminishing the otherwise unsightly aspect of a large metal filling.—W. STORER HOW, in *Transactions of American Dental Association.*

